# Correlation Between Nose-to-Chin Distance and Other Measurements Used to Determine Occlusal Vertical Dimension

**DOI:** 10.7759/cureus.60443

**Published:** 2024-05-16

**Authors:** ِAbdel Naser M Emam

**Affiliations:** 1 Prosthetic Dental Science, Faculty of Dentistry, Najran University, Najran, SAU

**Keywords:** prosthodontic, thumb, vertical dimension of occlusion, calliper, facial measurements, edentulous patients

## Abstract

Objectives: Accurate occlusal vertical dimension (OVD) establishment is an essential component of prosthodontic treatment. No accurate approach exists to determine the appropriate OVD for patients without posterior teeth. This study aimed to correlate the accuracy of the nose-to-chin distance with various facial measurements and thumb length in dentulous and edentulous Saudi patients.

Materials and methods: The participants comprised 100 fully dentulous Saudi male dental students aged 20-30 years (group 1) and 20 completely edentulous patients aged 60-70 years (group 2). We evaluated the correlations between the OVD (nose-to-chin distance) and the distance from the pupil to the corner of the mouth, the vertical length at midline of the nose (from subnasion to glabella), the distance from the outer canthus of one eye to the inner canthus of the other, twice the distance between the inner canthi, and the distance from the thumb tip to the index fingertip.

Statistical analysis: Spearman’s correlation and regression analysis were performed to analyze and assess the correlations between the clinically measured nose-to-chin distance and other parameters, with significance set at 0.05.

Results: In edentulous patients, the results showed a significant, strong, positive correlation between the nose-to-chin distance and the distance between the pupil and the mouth corner. In dentate subjects, a significant, strong, positive association was found between the nose-to-chin distance and the distance from the outer canthus of one eye to the inner canthus of the other. The linear regression analysis revealed that the distance between the pupil and the mouth corner in edentulous patients significantly predicted the OVD.

Conclusions: In both groups, the distance from the outer canthus of one eye to the inner canthus of the other and the distance from the pupil to the mouth corner were reliable and accurate for OVD measurements. These could be considered appropriate methods for OVD determination during full-mouth rehabilitation.

## Introduction

Posterior teeth loss can lead to neuromuscular mandibular instability, reduced masticatory efficiency, and loss of occlusal vertical dimension (OVD), the distance between the gnathion to the tip of the nose in the maximal intercuspal position [1]. Correcting the reduced OVD and posterior occlusal collapse necessitates oral rehabilitation. A crucial step for the prosthetic treatment involves establishing the optimum OVD. While various methods are reported, establishing the appropriate OVD remains difficult [2]. Establishing an accurate OVD for edentulous patients is essential when creating a complete denture, with flawed determinations of the OVD and centric relation that may result in treatment failure [3,4].

Mechanical and physiologic techniques have been used to find the OVD of completely edentulous patients. Mechanical methods have included using pre-extraction records, measurements, and ridge parallelism. Physiologic methods have included using the physiologic rest position, swallowing, and phonetics. Esthetics and patient-reported comfort may be added to these techniques [5,6].

Clinical OVD measurements often rely on determining the mandibular rest position, and these assessment procedures have been the subject of many investigations. However, phonetic procedures are still recommended and remain the commonest clinical method to evaluate the vertical dimension [7,8].

The height of an ear and one-third of the face are the same distances from the chin to the nose and the hairline to the eyebrows. The distance from the outer canthus of one eye to the inner canthus of the other also equals the height of the ear and of one-third of the face. Additionally, the face height (chin to hairline) equals the height of the hand, and the nose's length is the same as the thumb's (as well as the thumb's tip's distance from the index fingertip). Determining OVD through facial and body measurements is a common practice among many professionals (e.g., surgeons, artists, orthodontists, and morticians) [9].

Measuring facial anatomic landmarks has been a controversial method of deriving the OVD. The Willis device was developed to measure the distance from the lower border of the nasal septum to the lower border of the chin and from the outer canthus of the eye to the corner of the relaxed lip with the teeth in occlusion. While these measurements are theoretically equal, facial asymmetry calls their values into question [10].

Using facial measurements remains popular in clinical practice. In research, both the caliper and Willis gauge techniques are used [11]. The Willis gauge measures the distance from nasal septum to chin. Inaccuracies may occur with this method, caused by instrument angulations (especially for convex profiles and patients with mustaches, beards, short necks, full lips, or round chins) and gauge compression of the soft tissue under the chin and nasal septum. The caliper method measures the distance between points on the nasal tip and chin. It is influenced by soft tissue compression in the region of the skin markers. Selecting a method for measuring the OVD should consider the measurement reliability and accuracy, technique adaptability, equipment type and complexity, cost, and required time [12].

Some researchers have recommended the use of anthropometric measurements to determine the OVD. For example, correlated finger length with original OVD. Additionally, OVD and interpupillary distance within a 2-4 mm range have been shown to be significantly associated in males. These are simple, noninvasive, cost-effective methods that can be used in regular practice. However, further research is required to generalize correlations between facial measurements and OVD, such as variations across gender, ethnicity, and race [13,14].

Various methods have been suggested to measure the correct OVD [15,16]. Many current techniques perform these measurements in a physiologic rest position, which is difficult for older patients to achieve. Therefore, multiple facial and body measurements are used in lieu of a direct measurement, which can be employed to construct complete dentures [17].

This study aimed to determine the correlations between the accuracy of the nose-to-chin distance and (1) the distance from the pupil to the corner of the mouth, (2) the vertical length at midline of the nose (from subnasion to glabella), (3) the distance from the outer canthus of one eye to the inner canthus of the other, (4) twice the distance between the inner canthi, and (5) the distance between the thumb tip and the index fingertip with the fingers pressed together for recording the OVD. We hypothesized that a positive correlation would exist between the nose-to-chin distance and specific OVD measurements.

## Materials and methods

Participants selection

This study was conducted at the dental clinics of the College of Dentistry, Najran University. The Willis gauge measured the distance between the nasal septum and chin as a control. A modified caliper was used for the facial and finger measurements. This study was approved by the Research Ethics Committee of Najran University with approval number 202403-076-019326-043925. The participants were divided into two groups.

Group 1

One hundred fully dentulous Saudi male dental students, aged 20-30 years, were included, all of whom had fully erupted teeth. The exclusion criteria included a history of major orthodontic management, malocclusion, attrition of natural teeth, and posterior bite collapse due to posterior tooth loss.

Group 2

Twenty completely edentulous patients aged 60-70 years were selected from the clinic. The exclusion criteria include excessive soft tissue under the chin, muscle spasms or trismus, temporomandibular joint disorders, and abnormal jaw relationships.

All patients in group 2 were completely edentulous, with normal class I occlusion. The OVD was determined with the rest position. Pinhead-sized marks were made with an indelible pencil on adhesive tabs, one fixed to the nasal tip and another to the point of the chin. The distance between these two marks was measured with a caliper while the patient relaxed with closed lips. This measurement was repeated until at least three constant readings were obtained. The lower record block was trimmed until it occluded evenly with the upper block and the distance between the marks was that of the constant reading. A freeway space was then produced by removing 3 mm from the lower wax rim.

Measurements

**Figure 1:**
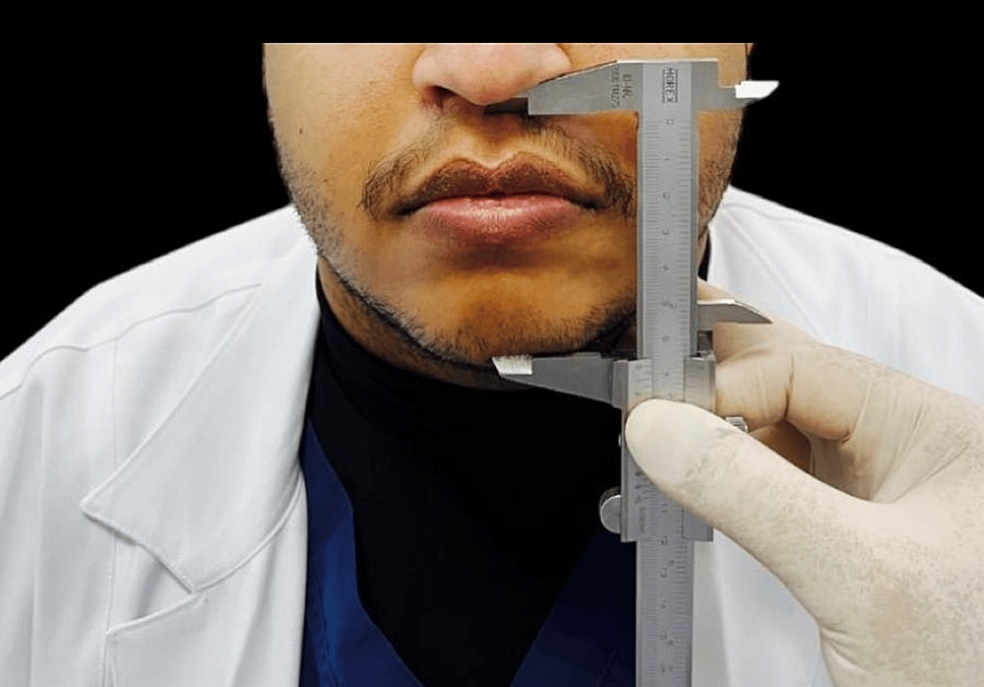
The nose-chin distance.

**Figure 2:**
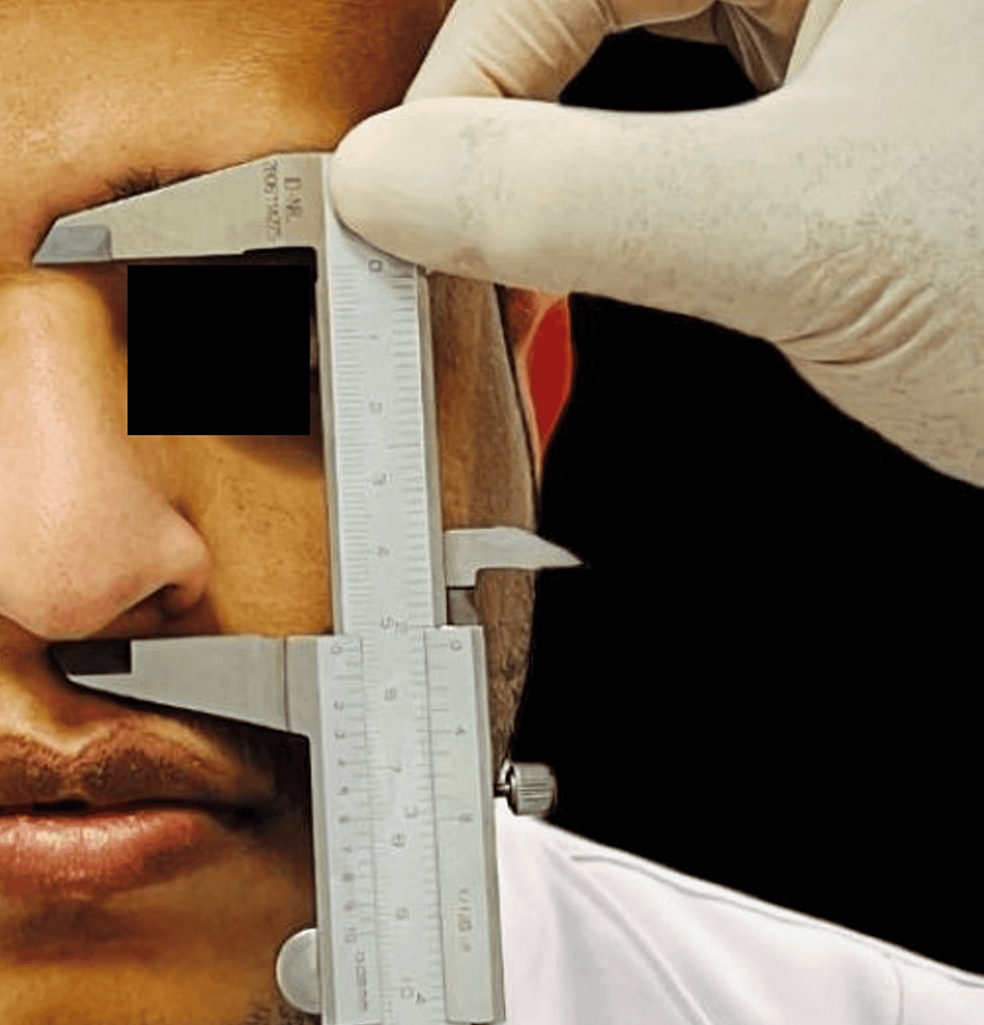
The vertical length of the nose at the midline.

**Figure 3:**
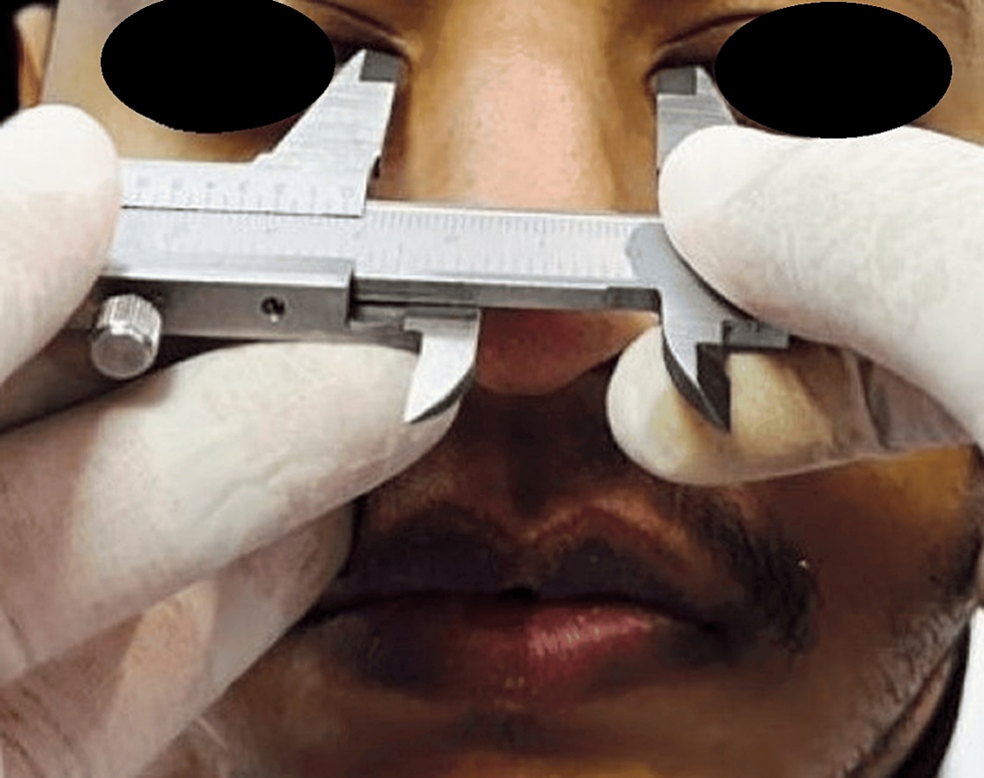
Twice the distance between the inner canthus of both eyes.

**Figure 4:**
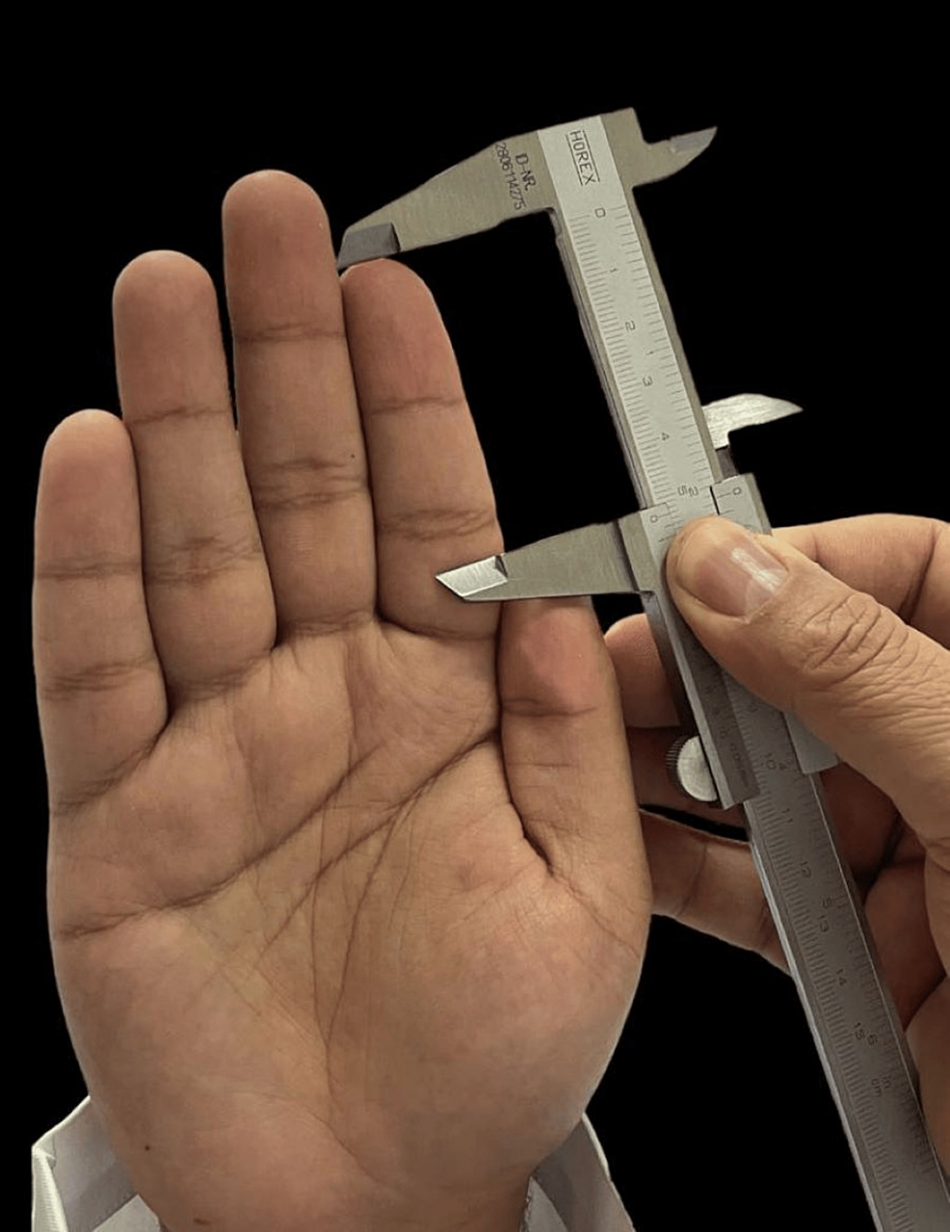
The distance between the tip of the thumb and the tip of the index finger.

**Figure 5:**
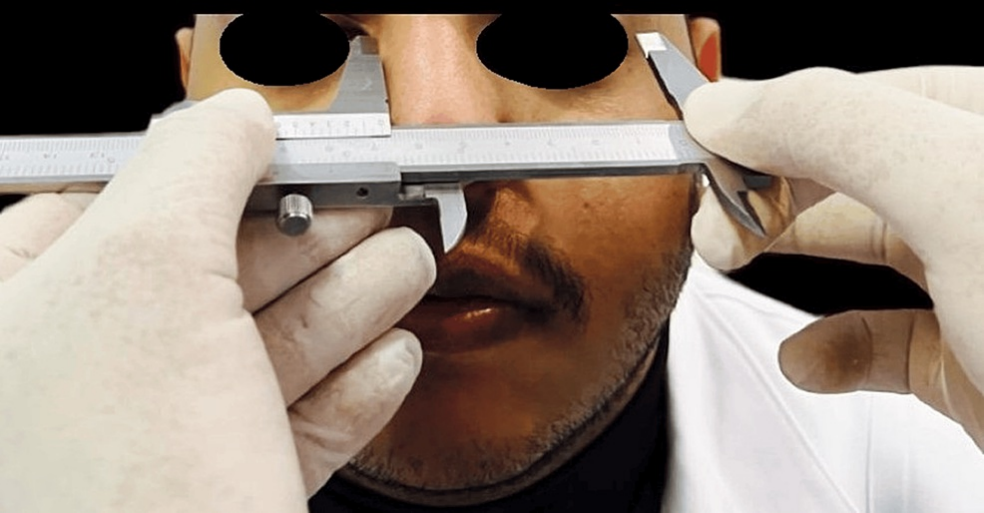
The distance from the outer canthus of one eye to the inner canthus of the other eye.

**Figure 6:**
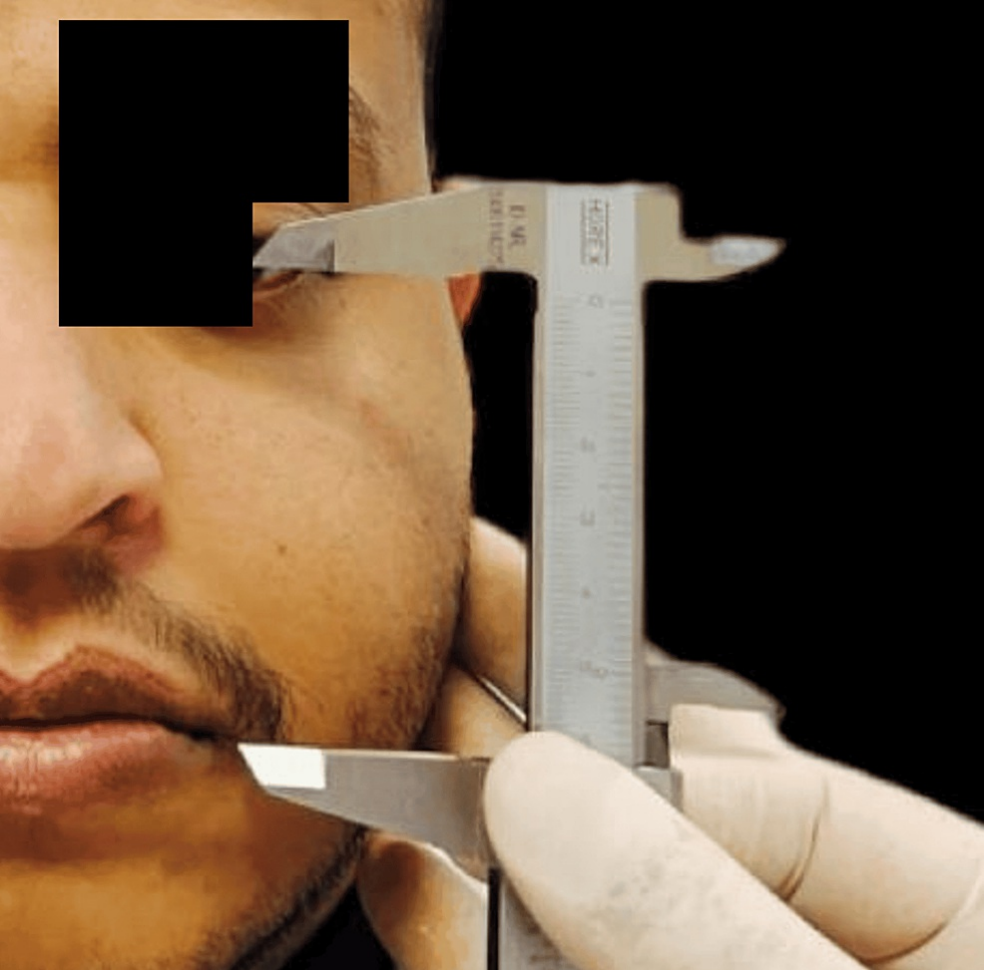
The distance from the pupil to the corner of the mouth.

**Figure 7:**
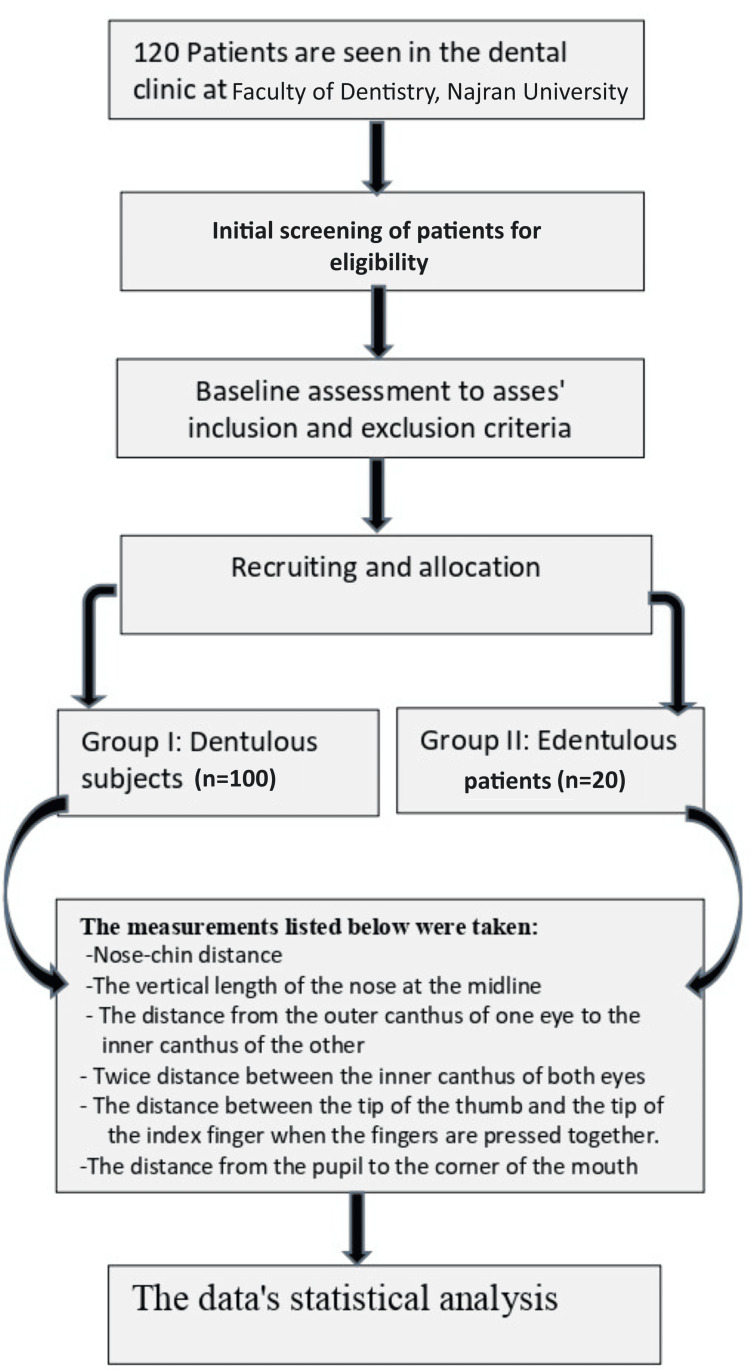
Flowchart shows how the study was designed.

The Willis gauge measured the distance from nasal septum to chin as a control (Figure 1). Measurements were taken at the occlusion of the recorded block for edentulous patients and at the centric occluding relation for dentulous participants. For both groups, a modified caliper was used to measure, on the right side, the vertical length of the nose at the midline (Figure 2), twice the distance between the inner corners of the eyes (Figure 3), the distance between the tip of the thumb and the index finger with the fingertip pressed together (Figure 4), the distance from the outer corner of one eye to the inner corner of the other eye (Figure 5) and the distance from the pupil to the corner of the mouth (Figure 6). All measurements were repeated until at least three constant readings were obtained. The measurements were tabulated and analyzed statistically (Figure 7).

Statistical analysis

The data were analyzed using SPSS version 23 (Armonk, NY: IBM Corp.). The Shapiro-Wilk test and visual inspection were conducted to assess the normality distribution of the variables in the dentate group. All tested variables, except for the nose length, significantly deviated from normality (W=0.979, p>0.05). In the edentulous patient group, all variables were normally distributed, except for nose-to-chin distance and finger length (W=0.865, p>0.05). Therefore, nonparametric tests were used to measure and summarize the statistics. Spearman’s correlation was employed to evaluate potential correlations between the clinically measured nose-to-chin distance and the other OVD measurements. Linear regression analysis was performed to develop a predictive model to determine the appropriate OVD measurements. All statistical tests were two-tailed and p>0.05 was considered statistically significant.

## Results

A statistically significant difference was found in the vertical midline nose length between edentulous and dentulous patients. Similarly, a statistically significant difference existed in the measurement of twice the distance between the inner canthi between edentulous and dentulous patients. A statistically significant difference was also found in the distance from the thumb tip to the index fingertip (finger length) between edentulous and dentulous patients (Table 1).

**Table 1:** Results of t-tests and descriptive statistics for measurements of OVD by edentulous (20 cases) and dentulous groups (100 cases). *P-value <0.05 was considered statistically significant. OVD: occlusal vertical dimension; Edent.: edentulous; Dent.: dentulous

Outcome	Mean Edent.	SD Edent.	Mean Dent.	SD	95% CI for mean difference	t	df	Sig.
Nose-chin distance	6.365	0.2996	6.403	0.5120	0.2078, 0.1318	0.451	44.744	0.654
Vertical length of the nose	5.865	0.4452	5.299	0.5289	0.3156, 0.8164	4.475	118	0.001*
Outer to inner canthus of other eye	6.330	0.2408	6.294	0.4346	0.1031, 0.1751	0.520	47.905	0.605
Twice the distance of both inner canthus	6.180	0.2949	5.714	0.4043	0.3090, 0.6230	6.025	35.021	0.001*
Index finger length	6.070	0.3011	5.848	0.5977	0.0415, 0.4025	2.466	54.292	0.017*
Pupil to the corner of the mouth	6.390	0.2511	6.381	0.4874	0.1401, 0.1581	0.121	52.675	0.904

No statistically significant difference was found in the nose-to-chin distance between edentulous and dentulous patients. Similarly, no statistically significant difference existed in the distance from the outer canthus of one eye to the inner canthus of the other between edentulous and dentulous patients. No statistically significant difference was found in the distance from the pupil to the mouth corner between edentulous and dentulous patients. The descriptive statistics in Table 1 show that edentulous patients exhibited higher OVD measurements compared to dentulous patients (Table 1).

In the dentate patient group, Spearman’s rank correlation was performed to evaluate the relationship between nose-to-chin distance and other OVD measurements. Table 2 shows a strong positive correlation between the nose-to-chin distance and the distance from the outer canthus of one eye to the inner canthus of the other (Spearman’s rank correlation r = 0.772, n = 100, p = 0.001). The nose-to-chin distance and the vertical length of the nose at the midline showed a moderately positive correlation (r = 0.405, n = 100, p = 0.001). A weak positive correlation existed between the nose-to-chin distance and the distance from the pupil to the mouth corner (r = 0.389, n = 100, p = 0.001). The nose-to-chin distance and the finger length showed a weak positive correlation (r = 0.387, n = 100, p = 0.001). A weak positive correlation existed between the nose-to-chin distance and twice the distance between the inner canthi (r = 0.201, n = 100, p = 0.045) (Table 2).

**Table 2:** Correlations and descriptive statistics for measurements of nose-chin distance and other measurements of OVD in the dentate patients (group 1) comprised 100 cases. *Correlation is significant at the 0.01 level. **Correlation is significant at the 0.05 level. OVD: occlusal vertical dimension

Variables	1	2	3	4	5	6
1. Nose-chin distance	1	-	-	-	-	-
2. Vertical length of the nose	0.405*	1	-	-	-	-
3. Outer to inner canthus of other eye	0.772*	0.323*	1	-	-	-
4. Twice distance of both inner canthus	0.201**	0.117	0.347*	1	-	-
5. Index finger Length	0.387*	0.100	0.258*	0.163	1	-
6. Pupil to the corner of the mouth	0.389*	0.348*	0.427*	0.150	0.262*	1
Median	6.500	5.300	6.300	5.800	5.800	6.400
SD	0.5120	0.5289	0.4346	0.4043	0.5977	0.4874
Scale min./max. values	5.2-7.5	4.1-6.5	5.5-7.5	4.6-6.4	4.5-7.1	5.5-7.5
Cronbach’s α	0.76					

In the edentulous patent group, Spearman’s rank correlation was also conducted to evaluate the relationship between the nose-to-chin distance and other OVD measurements. Table 3 shows a strong positive correlation between the nose-to-chin distance and the distance from the pupil to the mouth corner (r = 0.775, n = 20, p = 0.001). The nose-to-chin distance and the distance from the outer canthus of one eye to the inner canthus of the other showed a moderate positive correlation (r = 0.499, n = 20, p = 0.025). No correlation existed between the nose-to-chin distance and the vertical length at the midline of the nose (r = 0.407, n = 20, p = 0.075). The nose-to-chin distance and twice the distance between the inner canthi showed no correlation (r = 0.395, n = 100, p = 0.001). No correlation existed between the nose-to-chin distance and the vertical length at the midline of the nose (r = 0.331, n = 20, p = 0.154) (Table 3).

**Table 3:** Correlations and descriptive statistics for measurements of nose-to-chin distance and other measurements of OVD in the edentulous patients (group 2) comprised 20 cases. *Correlation is significant at the 0.05 level. **Correlation is significant at the 0.01 level. OVD: occlusal vertical dimension

Variables	1	2	3	4	5	6
1. Nose-chin distance	1	-	-	-	-	-
2. Vertical length of the nose	0.407	1	-	-	-	-
3. Outer to inner canthus of other eye	0.499^*^	0.230	1	-	-	-
4. Twice distance of both inner canthus	0.395	0.212	0.344	1	-	-
5. Index finger Length	0.331	0.508^*^	0.479^*^	0.587^**^	1	-
6. Pupil to the corner of the mouth	0.775^**^	0.519^*^	0.589^**^	0.662^**^	0.573^**^	1
Median	6.250	5.900	6.300	6.200	6.050	6.450
SD	0.2996	0.4452	0.2408	0.2949	0.3011	0.2511
Scale min/max values	6.0-7.0	5.0-6.8	6.0-6.9	5.4-6.6	5.5-6.8	6.0-6.9
Cronbach’s α	0.85					

The linear regression analysis model was fitted to the 20 edentulous patients (Table 4). The nose-to-chin distance was considered the dependent variable (outcome variable), and the other OVD measurements were considered explanatory variables. The correlations and regression results showed that the nose-to-chin distance was positively, strongly, and significantly correlated with the distance between the pupil and the mouth corner (F {5, 14} = 11.07, p = 0.001). The linear regression equation for the relationship between the nose-to-chin distance (Y) and the distance between the pupil and the mouth corner (X) is as follows: Y = 1.078X - 0.622. According to the model, measuring the distance between the pupil and the mouth corner in edentulous patients could significantly predict the OVD. The adjusted R^2^ = 0.726 reflects that the model explains 72.6% of the variance in the nose-to-chin distance. The value of the inflation factor result showed no evidence of multicollinearity between the variables. To determine the influence of each OVD measurement on the nose-to-chin distance, their coefficients were determined. The results showed that all other OVD measurements did not influence the nose-to-chin distance (Table 4).

**Table 4:** Regression analysis of the measurement of the nose-to-chin distance based on the other measurements of OVD in the edentulous patients (group 2). *Correlation is significant at the 0.05 level. OVD: occlusal vertical dimension; SE: standard error

Variable	B	SE	95% CI	t	Sig.
Pupil to the corner of the mouth	1.07*	0.262	0.51, 1.63	4.12	0.001*
Nose length	0.09	0.108	0.32, 0.13	0.90	0.379
Outer to inner canthus of other eye	0.37	0.236	0.13, 0.88	1.58	0.135
Double inner	0.17	0.162	0.51, 0.17	1.04	0.313
Length of finger	0.10	0.189	0.51, 0.29	0.56	0.582
Intercept	0.62	1.05	2.89, 1.64	0.58	0.56
Adjusted R^2^	0.726				
F	11.07				
Df	5, 14				

## Discussion

OVD restoration is essential in full-mouth rehabilitation because an inaccurate OVD compromises denture phonetics, esthetics, and functionality. Therefore, using repeatable procedures is critical for correct OVD determination [18,19]. For entirely edentulous patients, a precise assessment of the facial vertical dimension is crucial to the effectiveness of the prosthesis. The OVD cannot be changed by the dentist above the patient’s physiological needs. No precise scientific approach exists to identifying the right OVD. The dentist’s expertise, knowledge, discernment, and experience are essential factors. Recording the OVD through the measurement of facial anatomical landmarks has been a contentious approach [20].

In this study, various facial landmarks were used to examine the correlation between selected points and finger length and the correlations with OVD in dentulous and edentulous patients. Positive correlations were found, and selected points could be used as alternative measurement points for proper OVD measurements. Therefore, the study hypothesis was accepted. When comparing parameters between dentulous and edentulous patients, no significant difference was found in the nose-to-chin distance or the distance from the outer canthus of one eye to the inner canthus of the other between edentulous and dentulous patients, compared to the distance from the pupil to the mouth corner. However, significant relationships were found between the vertical length at midline of the nose (twice the distance between the inner canthi) and the distance between the thumb tip and index fingertip (finger length). These differences may be related to tissue changes occurring after tooth extraction and their effects on general health [21]. Similar to previous studies, the three following measurements showed a positive correlation: the distance between the mouth angles rest, the distance between the glabella and the subnasion, and the distance between the pupil center and a line projected laterally from the lips' median line [22,23].

A previous study observed in edentulous patients that the distance from the outer canthus of the eye to the mouth angle could be used as a highly accurate substitute for the nose-to-chin distance at rest (with dentures) [24], comparable to the work of Fenn et al. [25]. The distance from the outer canthus of the left eye to the mouth angle could also be used as a very highly accurate substitute for the nose-to-chin distance in occlusion, agreeing with Chou et al. [26].

Anthropometric measures are stable, patient-specific, repeatable, and generally do not change with age, making them appropriate landmarks [25]. Scarce data exists for the Saudi population. Therefore, this study aimed to examine the reliability of anthropological metrics used in the literature to predict OVD in Najran, Saudi Arabia. Finger length (thumb and index) was examined as an alternative to conventional methods, and a correlation was found with all measurements. Previous studies have used finger length for OVD measurements and reported that OVD was positively and significantly correlated with all parameters, landmarks, and selected measurement points. Despite the correlation between the selected points and finger length, the variations in readings mean that using finger length alone cannot be recommended. However, it could be used to confirm conventional and suggested techniques for OVD measurement [27].

Clinically, anthropometric measurements, such as facial measurements and finger lengths, can guide estimates of OVD and offer substantial prosthetic advantages. Their objective nature eliminates the guesswork of using subjective methods to determine OVD, such as swallowing and resting jaw position. Anthropometric methods to determine OVD are appealing because they are simple, dependable, inexpensive, and noninvasive. They require no radiography or complicated measurement gear and they produce repeatable results for future reference. These strategies take little time or experience to master [13].

A limited sample size and the inclusion of only participants with normal occlusion may be limitations of this study. Additionally, only males were included among the dentulous patients. Obtaining facial readings can be challenging, particularly for patients who have much soft tissue beneath the chin. Nonetheless, to further support the results, comparable analyses are recommended with larger sample sizes and diverse ethnic populations [22].

## Conclusions

The dentate patient group showed a strong positive correlation between the nose-to-chin distance and the distance from the outer canthus of one eye to the inner canthus of the other. The nose-to-chin distance and the vertical length at midline of the nose showed a moderate positive correlation. In the edentulous patent group, a strong positive correlation existed between the nose-to-chin distance and the distance from the pupil to the mouth corner. The nose-to-chin distance and the distance from the outer canthus of one eye to the inner canthus of the other showed a moderate positive correlation. The distance from the outer corner of one eye to the inner corner of the other eye is a reliable and accurate method for OVD measurement in dentate patients. While the distance from the pupil to the corner of the mouth is a reliable and accurate method for OVD measurement in edentulous patients. These measurements could be used as alternative methods when difficulty arises in using conventional methods. However, none can be used individually; the combination of several methods is recommended for accurate OVD measurement effectiveness.
